# Disruption of *Yarrowia lipolytica TPS1* Gene Encoding Trehalose-6-P Synthase Does Not Affect Growth in Glucose but Impairs Growth at High Temperature

**DOI:** 10.1371/journal.pone.0023695

**Published:** 2011-09-12

**Authors:** Carmen-Lisset Flores, Carlos Gancedo, Thomas Petit

**Affiliations:** 1 Department of Metabolism and Cell Signalling, Instituto de Investigaciones Biomédicas “Alberto Sols” CSIC-UAM, Madrid, Spain; 2 Université de La Réunion, Laboratoire de Chimie des Substances Naturelles et des Sciences des Aliments, Institut Universitaire de Technologie, Département Génie Biologique, Saint Pierre, France; Institute of Developmental Biology and Cancer Research, France

## Abstract

We have cloned the *Yarrowia lipolytica TPS1* gene encoding trehalose-6-P synthase by complementation of the lack of growth in glucose of a *Saccharomyces cerevisiae tps1* mutant. Disruption of *YlTPS1* could only be achieved with a cassette placed in the 3′half of its coding region due to the overlap of its sequence with the promoter of the essential gene *YlTFC1*. The *Yltps1* mutant grew in glucose although the *Y. lipolytica* hexokinase is extremely sensitive to inhibition by trehalose-6-P. The presence of a glucokinase, insensitive to trehalose-6-P, that constitutes about 80% of the glucose phosphorylating capacity during growth in glucose may account for the growth phenotype. Trehalose content was below 1 nmol/mg dry weight in *Y. lipolytica*, but it increased in strains expressing *YlTPS1* under the control of the *YlTEF1*promoter or with a disruption of YALI0D15598 encoding a putative trehalase. mRNA levels of *YlTPS1* were low and did not respond to thermal stresses, but that of *YlTPS2* (YALI0D14476) and *YlTPS3* (YALI0E31086) increased 4 and 6 times, repectively, by heat treatment. Disruption of *YlTPS1* drastically slowed growth at 35°C. Homozygous *Yltps1* diploids showed a decreased sporulation frequency that was ascribed to the low level of YALI0D20966 mRNA an homolog of the *S. cerevisiae MCK1* which encodes a protein kinase that activates early meiotic gene expression.

## Introduction

Trehalose, a non-reducing disaccharide formed by two glucose units, has important and varied functions in different organisms [1], [2]. In yeasts trehalose is synthesized by a two-step pathway [3]: first, trehalose-6-phosphate (T6P) is formed from glucose-6P and UDP-glucose by the enzyme T6P synthase (Tps1) encoded by the *TPS1* gene [4] and then dephosphorylated by a T6P phosphatase (Tps2) encoded by the gene *TPS2* [5]. Two other proteins without catalytic activity, Tps3 and Tsl1, appear to form a complex with Tps1 and Tps2 [6]. Mutations in the genes involved in trehalose biosynthesis affect glucose metabolism, morphology or virulence in yeasts and fungi [2], cause lethal phenotypes in insects and nematodes [7], [8] and are embryo lethal or affect inflorescence branching and other structures in plants [9]. In *Saccharomyces cerevisiae* or *Kluyveromyces lactis* mutations in the gene *TPS1* cause inability to grow in glucose [4], [10], [11]. This phenotype has been ascribed to the loss of the inhibitory effect of T6P on hexokinase [2], [12] and mathematical modelization of glycolysis has confirmed the importance of this control mechanism in *S. cerevisiae* [13]. The inhibition of hexokinase by T6P is widespread among yeasts [12], [14] but its strength is variable; the most inhibited hexokinase reported is that of the yeast *Yarrowia lipolytica* with a K_i_ of 3.5 µM [12], [15]. *Y. lipolytica* is a dimorphic yeast that separated early from the yeast evolutionary trunk [16]. It has attracted attention due to its ability to shift between a yeast and an hyphal form [17] to excrete organic acids [18], [19] and to its potential as host for expression of heterologous proteins [20]. *Y. lipolytica* is also being used as model to study physiological processes like lipid accumulation [21] or peroxisome biogenesis and pexophagy [22]. Differences in kinetic or regulatory properties of important *Y. lipolytica* enzymes [23], [24], [25] and in transcriptional regulation of some of its genes with respect to those found in *S. cerevisiae* [26], [27] have been described. Therefore due to the high sensitivity of *Y. lipolytica* hexokinase to T6P it appeared worthwhile to isolate the *TPS1* gene of this yeast and to analyze the effects of its disruption. The isolation of this gene presents also a potential technological interest as in *Aspergillus niger* the degree of expression of the *tpsA* gene that encodes T6P synthase, influences the rate of citric acid production [28], [29] and *Y. lipolytica* excretes this acid in some conditions [18], [19]. We report here that *Y. lipolytica* has a single gene encoding T6P synthase, that its disruption does not preclude growth in glucose but decreases sporulation efficiency and slows down growth at 35°C. In addition we report that disruption of *YlTPS3* abolishes the increase of trehalose observed during heat shock.

## Materials and Methods

### Strains and culture conditions

The yeasts strains used are shown in Table 1. *Y. lipolytica* was cultured in a synthetic medium with 0.17% yeast nitrogen base without amino acids and ammonium sulfate (Difco, Detroit, MI) and 0.1% glutamate pH 6. *S. cerevisiae* was cultured similarly but using ammonium sulfate as nitrogen source. Auxotrophic requirements were added at a final concentration of 20 µg/ml and 2% glucose was generally used as carbon source. Liquid cultures were shaken at 30°C. Sporulation medium was based in commercial V8 drink essentially as [30]. Freshly constructed diploid strains were patched on this medium and incubated at 23°C for up to two weeks. Sporulation was followed by malachite green staining [31]. Spores were recovered from sporulated cultures after digestion with Zymolyase 20T (Seikagaku Co.,Tokyo, Japan) and treatment with mineral oil. Cells in the hydrophobic phase were spread on selective plates and colonies isolated and checked for crossing ability and the relevant genes tested by PCR. Thermal stresses were done by transferring the yeast cultures from 30°C to a shaking water bath at 40°C or at 4°C and keeping them for the time indicated in each experiment. Temperature equilibration took place in less than five minutes.

**Table 1:** Yeast strains used in this work.

Strains of *Y. lipolytica*	Genotype	Reference
PO1a	MATA *leu2-270 ura3-302*	[34]
E129	MATA *lys11-23 ura3-302 leu2-270 xpr2-322*	C. Gaillardin (Grignon, France)
A1-5	MATB *met6*	S. Mauersberger (Dresden,Germany)
CJM 364	MATA *leu2-270 Δura3-302 tps1::YlURA3*	This work
CJM 645	MATB *met6*	From a cross A1-5×CJM 364
CJM 651	MATA *tps1::YlURA3 met 6*	From a cross A1-5×CJM 364
CLF 279	*tps1::YlURA3 leu2-270 met6* pCLF4	This work
CJM 613	MATA *leu2-270 ura3-302* pCLF5	This work
CJM 683	MATA *leu2-270 ura3-302 nth1::LEU2*	This work
CJM 402	MATA *tps1::YlURA3 lys11-23 ura3-302 leu2-270 xpr2-322*	This work. Derived from E129
CJM 649	MATB *tps1::YlURA3 leu2-270 ura3-302 met6*	From a cross A1-5×CJM 364
CJM 722	Diploid homozygous for *TPS1*	From a cross E129×A1-5
CJM 723	Diploid heterozygous *TPS1*/*tps1*	From a cross CJM 402×A1-5
CJM 724	Diploid homozygous *tps1*/*tps1*	From a cross CJM 402×CJM 649
CJM 748	MATA *tps3::LEU2 ura3 leu2-270*	This work
CJM 703	*tps1::YlURA3 nth1::YlLEU2 met 6*	From a cross CJM 649×CJM 683
CJM 667	MATA *leu2-270 ura3-302* pCLF8	This work
CJM 687	MATB *tps1::YlURA3 leu2-270 ura3-302 met6* pCLF9	This work

Strains of *S. cerevisiae*	Genotype	Reference
CJM 567	Wild type	J.M. Gancedo (Madrid, Spain)
CJM 486	*MAT*α *ade2-1 his311,15 ura3-1 leu2-3 112 trp1-1 tps1::HIS3*	[12]
CJM 391	*MAT*a *ade2-1 his311,15 ura3-1 leu2-3 112 trp1-1 tps1::HIS3* pCLF1	This work
CJM 397	*MAT*a *ade2-1 his311,15 ura3-302 leu2-3 112 trp1-1 tps1::HIS3* pCLF2	This work
CJM 767	*MAT*α *ade2-1 his311,15 ura3-1 leu2-3 112 trp1-1 tps1::HIS3* pCLF7	This work

Plasmids pCLF are described in Materials and Methods.

### Libraries, primers and plasmids

A *Y. lipolytica* cDNA library under the control of the *S. cerevisiae PGK1* promoter in plasmid pFL61 [32] and a genomic library of *Y. lipolytica* [33] were used. Yeast transformations were as described in [34] for *Y. lipolytica* and in [35] for *S. cerevisiae*. Primers used in PCR reactions are shown in Tables S1 and S2. All PCR products were sequenced to verify their identity.

The following plasmids for *S. cerevisiae* were constructed:

- pCLF1 carrying the *YlTPS1* gene was isolated from a cDNA library [32] by its ability to complement the lack of growth in glucose of a *S. cerevisiae tps1* strain.
- pCLF2, a centromeric plasmid that carries *YlTPS1*, was constructed as follows. The *Bam*HI fragment from plasmid pAN10 [36] carrying the promoter and the terminator regions of the *S.cerevisiae ADH1* was inserted into pRS316 [37] linearized with *Bam*HI. A 1.5 kb blunt-ended *Not*I fragment with *YlTPS1* from pCLF1 was inserted in the blunt-ended *Hin*dIII site of this plasmid.
- pCLF7 expresses *YlTPS3* under the control of the *ScADH1* promoter. *YlTPS3* (YALI0E31086) is annotated as an intron containing gene. Using the FirstChoice RLM-RACE Kit (Ambion) we checked the correctness of the ATG and the cDNA predicted sequence. Primer design to amplify the cDNA from genomic DNA was based on the fact that the first exon is only 23 bp long. Primer 1006 covers the first exon and the first 19 bp of the second exon; together with primer 1007 produced a PCR product of 3168 bp containing the cDNA of *YlTPS3*. This product was cloned in the pCR-Blunt vector (Invitrogen) and the resulting plasmid was digested with *Not*I and *Spe*I blunt-ended and cloned in pDB20 [38] in which the *URA3* marker had been substituted by *LEU2*. The cDNA of *YlTPS3* was sequenced again when introduced in this plasmid.

The following plasmids for *Y. lipolytica* were constructed

- pCLF3 carries a fragment of 5.4 kb that contains the *Y. lipolytica TPS1* gene and was isolated by screening a *Y. lipolytica* genomic library [33] with a *YlTPS1* probe.
- pCLF4 expresses *YlTPS1* under the control of its own promoter. It was constructed as follows: the *YlURA3* marker in plasmid pCL49 [26] was substituted by *YlLEU2* to give pCL49L. The *Sph*I-*Bam*HI fragment of pCL49L was replaced by a 2.8 kb *Hpa*I-*Bam*HI fragment from pCLF3 bearing the *YlTPS1* ORF and 1 Kb of upstream sequence.
- pCLF5 carries the coding region of *YlTPS1* (a 1.5 Kb *Not*I fragment) from plasmid pCLF1 under the control of the *YlTEF1* promoter in plasmid pCL49L.
- pCLF8 carries a fusion of the *YlTPS1* promoter to *lacZ*. A 1214 bp DNA fragment that includes the 14 initial amino acids of *YlTPS1* was obtained by PCR, using oligonucleotides 1010 and 1011 cloned into pGEM-T easy, digested with *Not*I and *Bam*HI and inserted into plasmid pINA354B [39] digested with the same enzymes. The resulting plasmid was linearized with *Apa*I to direct integration into the *YlLEU2* locus. Correct integration was checked by PCR and Southern analysis.
- pCLF9 carries the coding region of *ScTPS1* obtained by PCR using primers 1012 and 1013 in plasmid pCL49L.

### Disruption of *YlTPS1*

In two consecutive PCR reactions a I-*Sce*I restriction site and a deletion of 658 bp were created. Primers 1001 and 1002 with complementary ends including the recognition site of meganuclease I-*Sce*I, 1000 and 1003 were used. With pCLF3 as template and the mentioned primers two fragments corresponding to the 5′ and 3′regions of the disruption cassette were obtained. These products were used as template with primers 1000 and 1003 in a PCR reaction to obtain a disrupted *YlTPS1* copy. The product was cloned into pGEM-T easy, digested with I-*Sce*I and ligated to a 1.2 kb *YlURA3* fragment flanked by I-*Sce*I sequences from plasmid pINA-URA3-I-*Sce*I [40]. The 5268 bp fragment *Not*I was used to disrupt the chromosomal copy of *YlTPS1* gene.

### Disruption of *YlTPS3* (YALI0E31086)

A piece of 3611 bp containing the *YlTPS3* gene was obtained by PCR from genomic DNA and primers 1004 and 1005 and cloned in pGEM-T easy (Promega). The resulting plasmid was digested with *Xho*I, to remove a fragment of 758 bp, and ligated to a 2.1 kb band containing the *YlLEU2* gene from a *Nco*I digestion of the plasmid pINA62 [41] after filling-in the ends of both DNA segments. The chromosomal copy of *YlTPS3* was disrupted using a 4996 bp *Not*I fragment from the previous plasmid. Correct disruption was checked by PCR.

### Disruption of *YlNTH1* (YALI0D15598)

A fragment of 2049 bp containing YALI0D15598 [42] encoding a putative neutral trehalase (*YlNTH1*) was obtained by PCR using primers 1008 and 1009 and cloned in pGEM-T easy. The resulting plasmid was digested with *Nhe*I and *Xho*I to substitute an internal 816 bp fragment of the *NTH1* gene with a 2.1 kb fragment containing the *YlLEU2* gene from plasmid pINA62 [41] digested with *Nco*I. A 3342 bp *Not*I fragment from this construction was used to disrupt the chromosomal copy. Correct disruption was checked by PCR and Southern analysis.

### RT-qPCR

Total RNA from *Y. lipolytica* was extracted from flash-frozen cells [43] and processed as described therein. The quality of RNA was checked using the Agilent 2100 Bioanalyzer. The primers for RT-qPCR are shown in Table S2. They were checked for specificity using the Primer-BLAST from NCBI against the *Y. lipolytica* CLIB122 genomic sequence. Total RNA was reverse-transcribed into cDNA using the High Capacity RNA-to-cDNA Master Mix (Applied Biosystems). The cDNA levels were then analyzed using a LightCycler 480 from ROCHE and the LightCycler 480 SYBR Green I Master mix (Roche) with each primer at 250 nM. Each sample was tested in triplicate. After completion of the RT-qPCR melting-curve data were collected to verify PCR specificity, the absence of contamination and primer dimers. The gene YALI0F27533 (*ARP4*) was used to normalize the data.

### Extracts and assay of enzymatic activities

Cell free extracts were prepared by breaking the yeasts in buffer with glass beads in six cycles of 1 min of vortexing and 1 min on ice. The buffer was 20 mM imidazole pH 7, with the addition of 1 mM DTT and 1 mM EDTA when T6P synthase was assayed. The extract was centrifuged in the cold for 15 min at 13000 rpm in an Eppendorf table top centrifuge and the supernatant used for determination of enzyme activities. T6P synthase activity was determined by a two step method [3] in a mixture of 50 mM imidazole pH 7, 0.1 M KCl, 10 mM MgCl_2_, 1 mM EDTA, 10 mM glucose 6-P and 5 mM UDP-glucose. Samples were taken at different time intervals and boiled for 3 minutes to stop the reaction. The UDP formed was measured spectrophotometrically in a coupled assay with NADH, pyruvate kinase and lactate dehydrogenase. A blank without added glucose-6P was run in parallel for each sample. Hexokinase and glucokinase were assayed with glucose and fructose as in [15]. β-galactosidase was assayed in 80 mM sodium phosphate buffer, 8 mM KCl, 0.8 mM MgSO_4_, 40 mM β mercaptoethanol, and 2.6 mM 2-nitrophenyl β-D- galactopyranoside as described by Wallenfels [44]. Protein was assayed with the commercial BCA protein assay kit (Pierce).

### Trehalose determination

The cultures were harvested by centrifugation, washed with water and frozen until use. Trehalose was extracted with boiling water as described in [45]. Trehalose was determined enzymatically using the Trehalose assay Kit from Megazyme (Bray, Co. Wicklow, Ireland).

### Extraction and assay of intracellular metabolites

Yeast cells were quickly filtered through a Millipore AAWPO4700 and snap frozen in liquid nitrogen. The frozen pellets were dropped in boiling ethanol and treated as in [46]. Metabolites were determined spectrophotometrically as in [47]. For calculations, it was assumed that 1 g wet yeast has an intracellular volume of 0.6 ml [48].

**Nucleotide accession number.-** The sequence of the *YlTPS1* cDNA was deposited at the GenBank database with the accession number AJ011032.

## Results

### Cloning and characteristics of the *Y. lipolytica TPS1* gene

**Figure 1:**
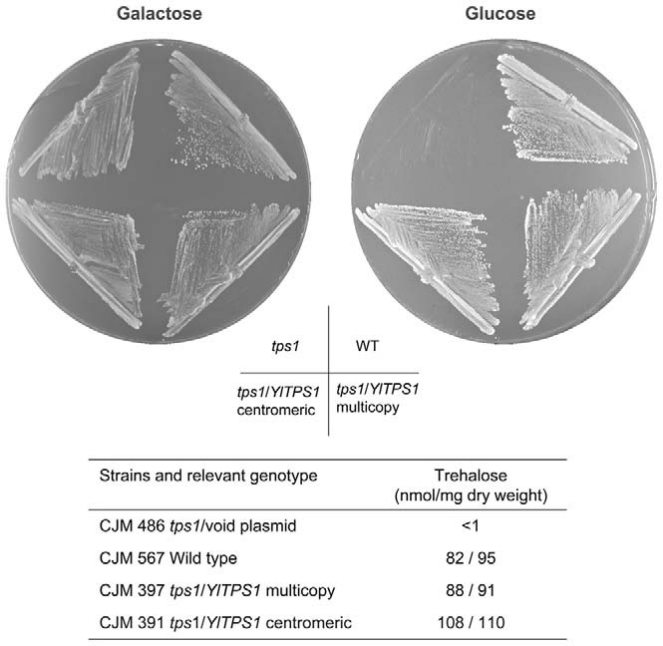
Phenotypic complementation of a *S.cerevisiae tps1* mutant by expression of the *YlTPS1* gene. The strains indicated were streaked on minimal medium with glucose or galactose as carbon sources and incubated at 30°C for 4 days. Trehalose content of the strains cultured with glucose, except the mutant *tps1* cultured in galactose, is given in the table (results of two independent cultures). The multicopy plasmid was pCLF1 and the centromeric one pCLF2.

**Figure 2:**
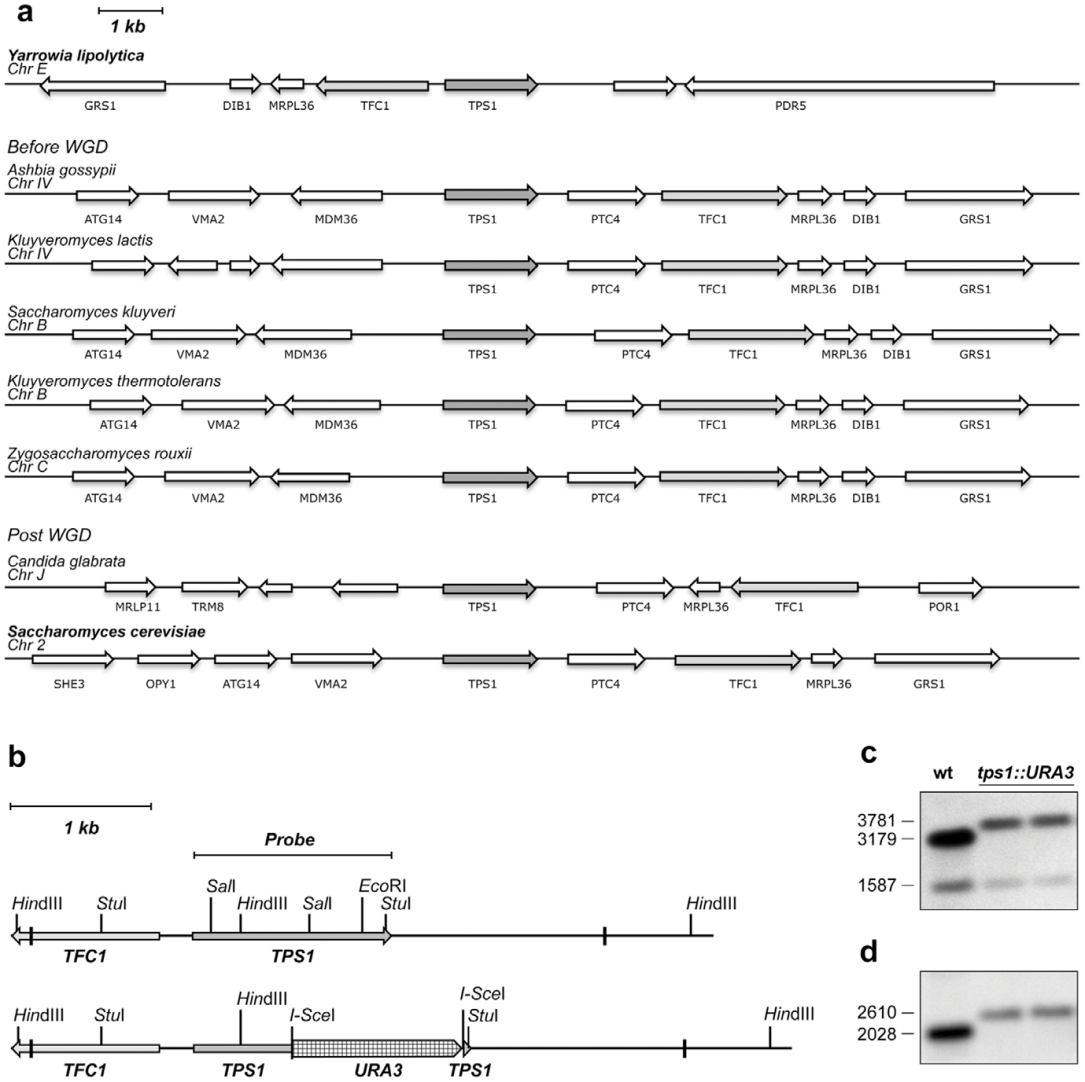
Relative positions of the *TPS1* and *TFC1* genes in several yeast species and disruption of the chromosomal copy of the *YlTPS1* gene. a) Diagram of the chromosomal neighborhood of *TPS1* in different yeasts species. Notice the unusual relative position of *TPS1* and *TFC1* in *Y. lipolytica*. The order and transcriptional direction of the genes correspond to the annotation in Génolevures [42] except for *S. cerevisiae* for which the annotation of SGD was used [90]. Names of genes appear below each arrow, arrows without name indicate genes of unknown function. Chromosome designation is also indicated. WGD, whole genome duplication. b) Scheme of the chromosomal region around *YlTPS1* and its disruption. The location of the coding region of *YlTFC1* is also shown. The I-*SceI* sites flanking the *URA3* gene were introduced by PCR as described in Materials and Methods. Short vertical bars indicate the piece of DNA used for the chromosomal disruption. c, d) Southern analysis of two different disruptants: genomic DNA was digested with *Hin*dIII (c) or *Stu*I (d) and hybridized with the indicated probe. The sizes of the DNA bands in bp are shown. Attempts to disrupt *YlTPS1* placing *YlURA3* between the *Sal*I sites or between *Hind*III and *Eco*RI were unsuccessful.

We used the phenotypic complementation of the absence of growth in glucose of a *S. cerevisiae tps1* mutant [2], [49] to isolate the *TPS1* gene from *Y. lipolytica*. We transformed a *S. cerevisiae tps1* mutant strain with a cDNA library from *Y. lipolytica* under the control of the *S. cerevisiae PGK1* promoter (see Materials and Methods) and selected transformants that grew on glucose. Plasmid pCLF1 isolated from different transformants carried a DNA insert of about 1.5 kb whose DNA sequence revealed an ORF encoding a putative protein of 486 amino acids that shares about 70% identity with different fungal T6P synthases. This *Y.lipolytica* cDNA inserted either in a multicopy or in a centromeric plasmid not only complemented the glucose negative phenotype of the *S. cerevisiae tps1* mutant but also restored its ability to synthesize trehalose (Figure 1). These results indicate that the DNA cloned encodes a *bona fide* Tps1 protein from *Y. lipolytica* that is functional in *S. cerevisiae*. We will refer to the gene encoding that protein as *YlTPS1*. The 1 kb upstream region of *YlTPS1* contains a CCCCT motif [50] that in *S. cerevisiae* is implicated in heat and other stress-controlled transcription [51], [52], [53], [54]. About 245 bp upstream of the ATG of *YlTPS1* we found the ATG of an ORF whose transcription runs divergent to that of *YlTPS1* (Figure 2a). The protein putatively encoded by this ORF is highly similar to the *S. cerevisiae* Tfc1, one of the two DNA-binding subunits of the yeast transcription factor TFIIIC. Part of the *TFC1* promoter likely overlaps with the coding sequence of *YlTPS1*. The relative position of these ORFs in *Y. lipolytica* is different from that found in other *Hemiascomycetes* in which *TPS1* appears in a synteny block that covers at least nine genes (Figure 2a). The sequence of the *TPS1* promoter region of the widely used PO1a strain exhibited a GT deletion at −60 and a change C/T in position −314 relative to ATG with respect to the sequence that appears in Génolevures [42].

### Disruption of the chromosomal copy of *YlTPS1* does not affect growth in glucose

**Figure 3:**
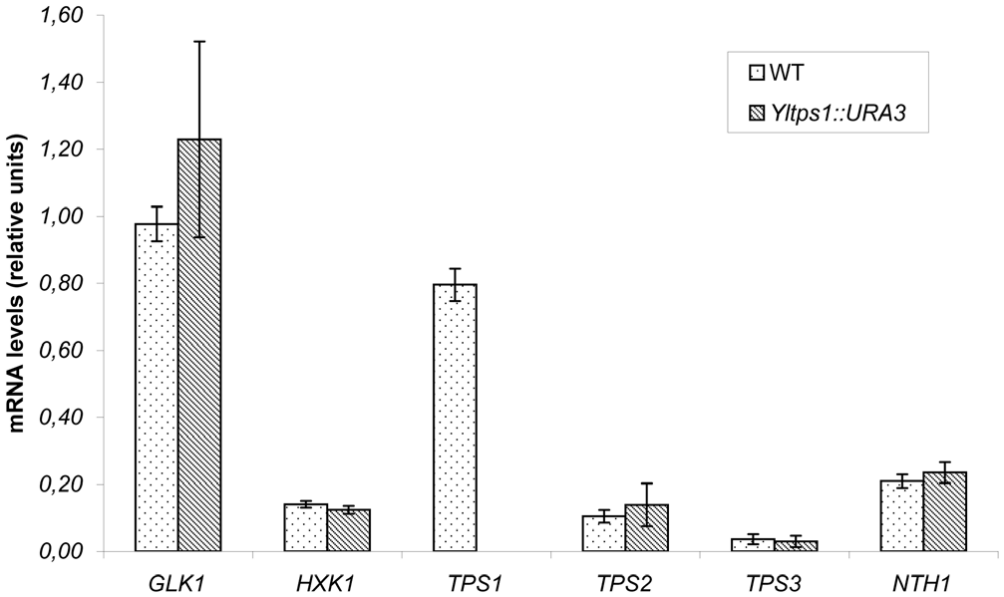
mRNA levels corresponding to genes encoding glucose phosphorylating enzymes and proteins related to trehalose metabolism in *Y. lipolytica*. Yeasts strains CJM 645, wild type, and CJM 651, *Yltps1*::*URA3*, were grown in minimal medium- glucose as indicated in Materials and Methods. mRNA levels were quantified by RT-qPCR as described in Materials and Methods. Three independent cultures were analyzed and three technical replicas were done for each run. Expression of each gene was normalized to that of the *YlARP4*. The columns represent the mean values of the three biological experiments with bars indicating the standard deviation. The genes studied encode the following proteins: *GLK1*, glucokinase; *HXK1*, hexokinase; *TPS1*, trehalose-6-P synthase; *TPS2*, trehalose-6-P phosphatase; *TPS3*, subunit of the trehalose synthase complex; *NTH1*, neutral trehalase.

Lack of growth in glucose of *S. cerevisiae tps1* mutants has been attributed to loss of inhibition by T6P of hexokinase 2 [12] the glucose phosphorylating enzyme expressed during growth in this sugar [55], [56]. Since T6P inhibition of *Y. lipolytica* hexokinase is the highest reported (K_i_ 3.5 µM) [12], [15] we studied the effect of the disruption of *TPS1* in this yeast. Attempts to disrupt *YlTPS1* placing the disruption cassette after nucleotides 188 or 406 after the ATG failed, only when it was displaced to nucleotide 710 a correct disruption was obtained (Figure 2 b,c,d). We attribute the failures with the disruption cassettes located in the 5′region of the coding sequence to interference with the expression of the neighbouring *TFC1* gene (Figure 2a). Lack of *TFC1* is lethal in *S. cerevisiae* [57] and in *Schizosaccharomyces pombe* [58] and this is likely to be the case also in *Y. lipolytica*. The *YlTPS1* disruptants grew in glucose in contrast with the behavior of the *tps1* mutants of *S. cerevisiae*. The distinct phenotype could be caused by a difference in the glucose phosphorylating equipment between the two yeasts, by a lack of significant activity of Tps1 or both. We found that in glucose grown cultures of *Y. lipolytica* expression of the gene YALI0E15488 encoding a glucokinase (Flores and Gancedo, unpublished results) exceeded that of the gene encoding hexokinase (Figure 3). Also enzyme measurements showed that glucokinase constitutes the main phosphorylating activity in *Y. lipolytica* (Table 2). Since glucokinase is insensitive to inhibition by T6P [12], [15], the growth in glucose of the *Yltps1* mutant may be explained by the scarce contribution of hexokinase to the glucose phosphorylating activity. Moreover, disruption of *YlTPS1* slightly decreased the proportion of hexokinase activity (Table 2). Concentration of hexose mono or bis- phosphates and ATP were not affected with respect to that of a wild type during growth in glucose (Table 3) in contrast to what happens in a *S. cerevisiae tps1* mutant which accumulates those compounds and loses ATP upon glucose addition. This result is consistent with a lack of control of the glycolytic flux by T6P in *Y. lipolytica*. The *YlTPS1* disruptants showed a slightly shorter duplication time than the wild type (Wt 149±4 min, *tps1* 139±4 min, *tps1*/pCLF5 151±8 min, means of four experiments) (Figure S1). No immediate explanation can be provided for this difference.

**Table 2:** Phosphorylating activity on glucose and fructose and activities of hexokinase and glucokinase in *Y. lipolytica*.

Strain and relevant genotype	Phosphorylating activity (mU/mg protein)		Specific activity (mU/mg protein)	
	Glucose	Fructose	Hexokinasea)	Glucokinasea)
CJM 645 *YlTPS1*	203±20	68±19	49 [24]	154 [76]
CJM 651 *Yltps1*	208±15	47±6	34 [16]	174 [84]

Strains were cultured in minimal medium glucose as indicated in Materials and Methods. Extracts and assay of phosphorylating activities were as described in Materials and Methods.

a) Hexokinase and glucokinase activities were calculated from the phosphorylation results with glucose and fructose using a Fructose/Glucose phosphorylation ratio of 1.4 for hexokinase ([92], our unpublished results). Figures in brackets indicate the percentage of the corresponding activity in the extract. Results are the mean values ± the standard deviations of four independent cultures.

**Table 3:** Concentration of metabolites (mM) in a WT and Yltps1 strains during growth in glucose.

Strains and relevant genotype	Glucose-6-P	Fructose-6-P	Fructose-1,6-P_2_	Trioses phosphate	ATP
CJM 645 *YlTPS1*	0.18±0.06	0.04±0.01	0.33±0.02	0.08±0.02	0.47±0.10
CJM 651 *Yltps1*	0.24±0.01	0.06±0.01	0.28±0.01	0.09±0.01	0.54±0.08

Yeast strains were cultivated in minimal medium glucose and sampled in exponential phase of growth. Metabolite extraction and quantification were done as described in Materials and Methods. Results are the mean values ± the standard deviations of three independent cultures.

### Levels of trehalose in *Y. lipolytica* are low but increase upon disruption of a gene encoding a putative trehalase or after heat shock

Levels of trehalose in *Y. lipolytica* grown in glucose up to stationary phase or in glycerol were below 1 nmol/mg dry weight. A possible explanation for this result could be that the genes encoding the trehalose biosynthetic pathway enzymes were not expressed during growth in glucose, therefore we measured the expression of those genes in *Y.lipolytica*. In the Génolevuress database [42], YALI0D14476 is annotated as similar to *S. cerevisiae TPS2* and YALI0E31086 shows the highest homology with *S. cerevisiae TPS3/TSL1*. All these genes were expressed during growth in glucose although the levels of *YlTPS2* and *YlTPS3* were low when compared to that of *YlTPS1* (Figure 3). Although a western blot analysis of a fusion of Tps1 to the HA epitope indicated that the protein was expressed during growth in glucose (results not shown) its activity was very low (Table 4). This low activity could be an additional cause of the lack of effect of the *YlTPS1* disruption on the growth in glucose. Expression of *YlTPS1* under the control of the strong *YlTEF1* promoter increased trehalose content and allowed detection of Tps activity which was almost undetectable in the wild type strain (Table 4). This result suggested that the low trehalose level was due to a low activity of the biosynthetic pathway. In addition to a low synthesis the low trehalose content could be due to the activity of a trehalase. We identified a gene, YALI0D15598, as the only sequence in the *Y. lipolytica* Génolevures database [42] that shows homology with the *S.cerevisiae NTH1* or *NTH2* genes encoding neutral trehalases. Measurements of *YlNTH1* mRNA showed that it was expressed during growth in glucose (Figure 3). We disrupted *YlNTH1* and measured an increased trehalose content in the resulting mutant. Such increase was dependent on the activity of Tps1 since in a double mutant *nth1 tps1* no trehalose was detected (Table 4). In plants also, inhibition of trehalase allows measurement of otherwise undetectable trehalose levels [59], [60], [61].

**Table 4:** Trehalose content and Tps1 activity in different strains of *Y. lipolytica*.

Strain and relevant genotype	Trehalose	Trehalose-6-P synthase
	(nmol/mg dry weight)	(mU/mg protein)
CJM 645 *TPS1*	<1	<5
CJM 613 *TPS1*pCLF5	8.9±2.1	55±5
CJM 683 *TPS1 nth1*	18±2	n.d.
CJM 703 *nth1tps1*	<1	n.d.

Yeasts were grown in minimal medium glucose; trehalose and trehalose-6-P synthase were assayed as described in Materials and Methods. Results are the mean values ± the standard deviations of three independent cultures. n.d., not determined.

**Figure 4:**
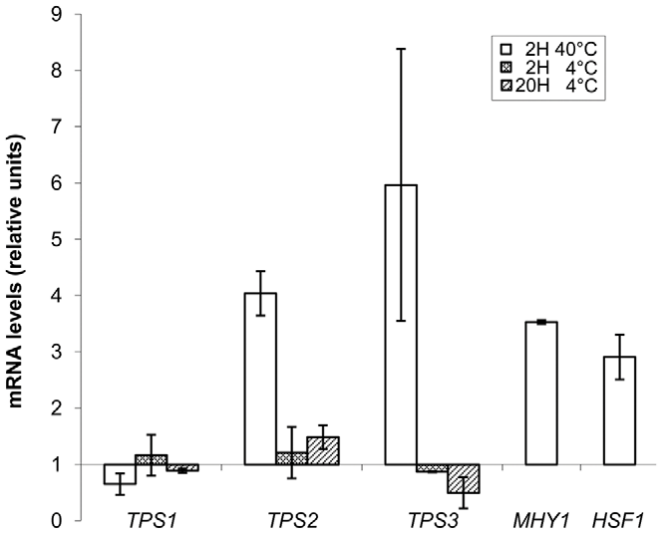
Levels of mRNA corresponding to genes *YlTPS1*, *YlTPS2*, *YlTPS3*, *MHY1* (YALI0B21582) and *HSF1* (YALI0E13948) during thermal stresses. A sample of an exponentially growing culture of strain CJM 645 in minimal medium glucose (time zero) was taken before its transfer to 40°C or 4°C for 2 or 20 hours. mRNA levels were quantified by RT-qPCR as described in Materials and Methods. Two independent cultures were analyzed and three technical replicas were done for each run. Expression of each gene was normalized to that of *YlARP4*. Values are shown relative to that of time zero. The columns represent the mean values of the experiments with bars indicating the standard deviation.

**Figure 5:**
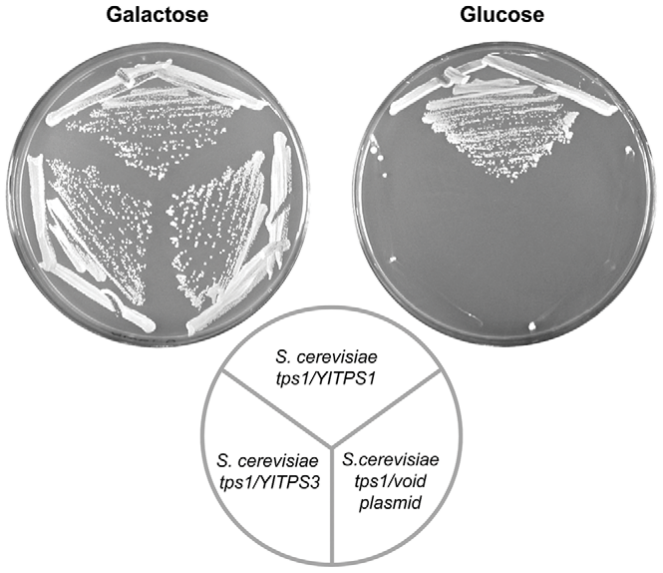
Lack of complementation of *Sctps1* by *YlTPS3*. The *S. cerevisiae tps1* mutant (CJM 486) transformed with plasmids pCLF1 (*YlTPS1*), pCLF7 (*YlTPS3*) and pDB20 (void) were streaked on minimal medium with glucose or galactose and incubated at 30°C for 4 days.

Heat shock increases trehalose in several yeasts [52], [62]. In *Y. lipolytica* a heat shock at 40°C for 2 h increased trehalose, although the final value varied depending on the strain (from 7 to 20 nmol/mg dry weight). This treatment was considered a strong one as this yeast does not grow at temperatures over 35°C. We measured the levels of mRNA corresponding to the genes related with trehalose metabolism after heat treatment (Figure 4). Levels of *YlTPS1* mRNA did not increase in spite of the presence of one CCCCT sequence in its promoter. Measurements of β-galactosidase produced from a fusion of the *YlTPS1* promoter to *E. coli lacZ* were consistent with this result (34±3 mUnits/mg protein at time 0 vs 39±4 mUnits/mg protein after 2 h, four independent experiments). Relative abundance of mRNAs corresponding to *YlTPS2* and to *YlTPS3* increased 4 and 6 times respectively with the heat treatment. The higher increase of *YlTPS3* suggested an important role for this gene in the heat response. A disruption of *YlTPS3* abolished trehalose accumulation upon heat treatment raising the question that Tps3 could be another T6P synthase an hypothesis that cannot be ruled out due to the similarity between the *YlTPS1* and *YlTPS3* sequences. This possibility was made unlikely by the absence of trehalose in a *Yltps1* mutant after heat shock and by the lack of complementation of the glucose negative phenotype of a *S. cerevisiae tps1* mutant by the *YlTPS3* cDNA (Figure 5). We suggest that *Yl*Tps3 is necessary to maintain the stability of the trehalose synthase complex during heat shock in *Y. lipolytica*. This reveals a difference with *S. cerevisiae* where the absence of Tps3 does not affect trehalose content during heat shock [6].

**Figure 6:**
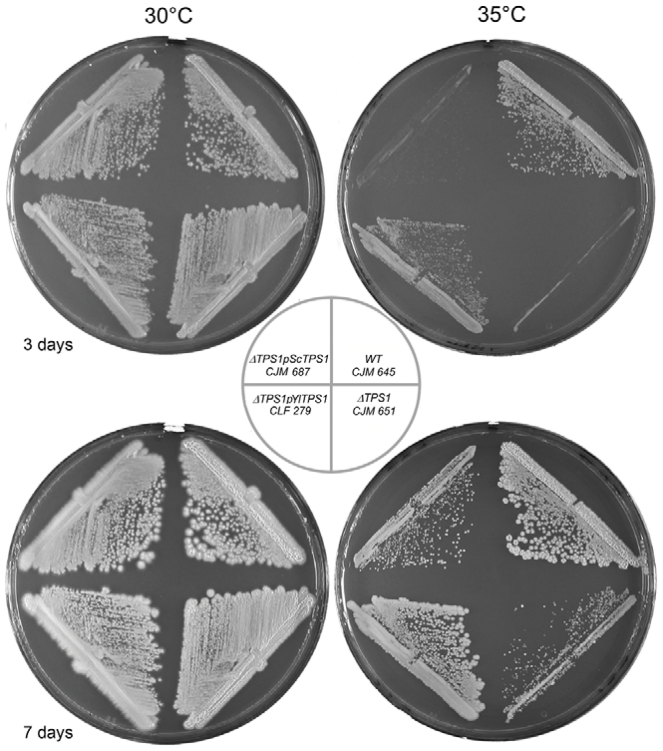
Effect of *YlTPS1* disruption on growth at 35°C. The strains were streaked on minimal medium glucose plates, incubated at the indicated temperatures and pictures taken 3 and 7 days after inoculation.

Disruption of *YlTPS1* severely decreased growth at 35°C, only small colonies were visible after 7 days at this temperature (Figure 6). A plasmid carrying *YlTPS1* restored a wild type phenotype. The *S.cerevisiae TPS1* gene slightly improved growth of the *Yltps1* mutant at 35°C.

Treatment of *Y.lipolytica* at 4°C during 2 or 20 hours did not modify significantly the mRNA levels corresponding to the genes of the trehalose biosynthetic pathway (Figure 4), in contrast with the behavior of *TPS1* and *TPS2* in *S. cerevisiae* whose expression increase upon a treatment below 10°C [63].

In *S. cerevisiae* transcription factors Hsf1 and Msn2/4 are implicated in the response to heat shock and other stresses [52], [64], [65]. In *Y. lipolytica* the protein Mhy1 (YALI0B21582p) shows high similarity in its zinc finger domain to that of Msn2/4 and also binds STRE sequences [66]. A BLAST search of the *Y. lipolytica* database for genes encoding homologues of *ScHSF1* yielded gene YALI0E13948. Levels of mRNA corresponding to those genes increased about 3 times after heat shock (Figure 4) consistent with their possible implication in heat shock regulated processes.

### Disruption of *YlTPS1* impairs sporulation

**Figure 7:**
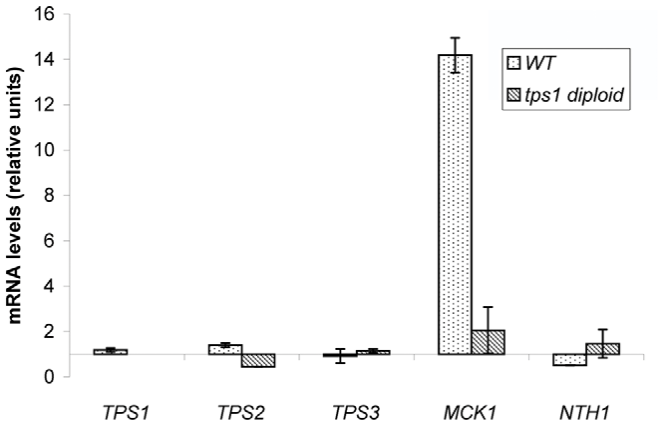
Levels of mRNA corresponding to several genes during sporulation. A wild type diploid and an homozygous *tps1* diploid were transferred from minimal medium glucose to V8 sporulation medium (see Materials and Methods). Samples were taken from the initial culture on glucose (time zero) and after 8 days in V8. mRNA levels were quantified by RT-qPCR as described in Materials and Methods. Three independent sporulation experiments were analyzed and three technical replicas were done for each run. Expression of each gene was normalized to that of *YlARP4*. Levels of mRNA are shown relative to that of time zero. The columns represent the mean values of the experiments with bars indicating the standard deviation.

When *Y. lipolytica* diploids homozygous for the *tps1* mutation (CJM 724) were placed in sporulation conditions the sporulation frequency was reduced with respect to that of wild type (CJM 722) or heterozygous *TPS1*/*tps1* (CJM 723) diploids. A similar behaviour in *tps1/tps1* diploids in *S. cerevisiae* [67], [68] was ascribed to a decreased expression of *MCK1*, a gene that stimulates expression of *IME1* which encodes a transcriptional activator of sporulation [68]. Based on sequence homology we identified an ORF YALI0D20966 which exhibits 41% identity and 59% similarity with *S. cerevisiae MCK1*. We have measured the levels of mRNA corresponding to YALI0D20966 and to the genes implicated in trehalose metabolism both in a wild type diploid and in one homozygous for the *tps1* mutation in sporulation conditions. After 8 days on sporulation medium the level of YALI0D20966 mRNA in a wild type was increased about 15 times while in a homozygous *tps1*/*tps1* strain it reached a maximum of 3 fold (Figure 7). This behaviour parallels that of *MCK1* in *S. cerevisiae* suggesting an implication of YALI0D20966 in sporulation in *Y. lipolytica*. Levels of RNA corresponding to *YlTPS1*, *YlTPS2*, *YlTPS3* or *YlNTH1* did not vary significantly in either strain under the same conditions.

## Discussion

We have isolated and characterized the gene encoding trehalose-6-P synthase from the dimorphic yeast *Y. lipolytica*. The identity of the gene is supported by the increase in trehalose and Tps activity in *Y. lipolytica* when the gene is expressed under the control of a strong promoter, the restoration of the growth in glucose and trehalose content to a *S. cerevisiae tps1* mutant, and by sequence similarity to T6P synthases from other organisms. Although other sequences with high similarity to Tps1 could be identified in the *Y. lipolytica* Génolevuress database [42] the only one active in trehalose synthesis when expressed in *S. cerevisiae* was *YlTPS1*. In most yeasts and fungi there is only one gene encoding a protein with Tps activity although in *Aspergillus niger* and *A. fumigatus* [69], [70] two T6P synthases with high sequence homology but with different roles in the physiology of the organism have been described. The chromosomal close vicinity of *YlTPS1* and *YlTFC1* deserves attention. A short intergenic region has been taken as indicative of sharing certain regulatory elements of the genes in question [71], [72]. Assuming an average promoter length of about 1 kb [72] the promoter of *TFC1* will overlap with the coding sequence of *YlTPS1* and viceversa. This neighborhood together with their opposite transcription direction explains the failures to disrupt *YlTPS1* with insertions located near the 5′end of its coding sequence as these may interfere with expression of *YlTFC1* and result in lethality. Although closely located pairs of genes with divergent transcription tend to be conserved in evolution [72] *TPS1* and *TFC1* are separated in other yeast species all along the Hemiascomycetes phylogenetic three [16]. This separation may have allowed the evolution of mechanisms linking the expression of *TPS1* to multiple signals as it is the case in other yeasts.

Deffects in the trehalose biosynthetic pathway produce a variety of effects in different organisms like bacteria [73] yeasts [2], plants [74] insects [7] or nematodes [8]. In *S. cerevisiae* loss of the control on hexokinase activity by T6P is one of the causes of the lack of growth of a *tps1* mutant in glucose [12], while in *S. pombe* whose hexokinases are not sensitive to T6P [75] this phenotype is not observed for a similar mutation [76]. The growth in glucose of the *tps1* mutant of *Y. lipolytica* whose hexokinase is highly sensitive to T6P [12], [15] could be explained by the presence of a T6P insensitive glucokinase constituting roughly 80% of the glucose phosphorylating capacity of this yeast. A similar explanation might also apply for the phenotype described for a *tps1* mutant of *Hansenula polymorpha* [14] a yeast in which glucokinase and hexokinase are present during growth in glucose [77]. The levels of metabolites in the disrupted strain are in accordance with the lack of effect of the mutation on the growth in glucose. A slight increase in ATP concentration was measured in the *Yltps1* mutant, a finding that parallels the results obtained for a *tpsA* disruptant of *A. nidulans* [78]. With our current knowledge no clear explanation for these results can be advanced. Due to the “turbo design” of glycolysis [13] a regulation of the initial steps of the pathway is necessary. In mammals glucose-6-phosphate controls hexokinase [79] and in *S. cerevisiae* T6P plays a similar role. Yeasts or fungi in which lack of T6P does not affect growth in glucose [14], [78] shall possess other mechanisms to regulate the first steps of glycolysis. It may be asked for the significance of the T6P inhibition of hexokinase in those organisms in which it appears not to play a significant role in the control of glycolysis. One possibility is that it may serve to control a yet unrecognized function of hexokinase, another one is that it is a consequence of the protein structure shared by most hexokinases and that organisms with a high glycolytic flux have taken advantage of it to control the first irreversible step of glucose metabolism. In *Y. lipolytica* differences in kinetic and regulatory properties of important glycolytic enzymes like phosphofructokinase [23] or pyruvate kinase [24] indicate that this yeast regulate glycolysis differently from *S. cerevisiae*.

The decrease in sporulation observed in homozygous *tps1* diploids parallels findings with *tps1* mutants in other fungi like *S. cerevisiae* [67], [68], [80] *Cryptococcus neoformans* [81] or *Stagonospora nodorum* [82]. In *S. cerevisiae*, the defect has been adscribed to a low expression of *MCK1* an inducer of the gene *IME1* whose expression triggers sporulation [68]. The low level in *Yltps1* diploids of mRNA corresponding to gene YALI0D20966 that appears to be the *Y. lipolytica* homolog of *ScMCK1* will suggest a similar mechanism for the decreased sporulation in this yeast and that the relationship between *TPS1* and sporulation was already present in an ancient yeast like *Y. lipolytica*.

Trehalose in *Y. lipolytica* in different conditions was below 1 nmol/mg dry weight. Disruption of a gene encoding a putative neutral trehalase or overexpression of *YlTPS1* increased trehalose content. A similar situation occurred in vascular plants in which trehalose was thought to be absent; incubation with validamycin A, an inhibitor of trehalase, showed the existence of the disaccharide [59], [60]. Hydrolysis of trehalose by trehalase and a low level of Tps1 activity may be responsible for the low levels of the sugar in *Y. lipolytica*. It could be speculated that the main role of Tps1 is to provide T6P as intermediate for pathways different from trehalose synthesis. Some bacteria produce biosurfactants or glycolipids that require T6P for their synthesis [83], [84]. *Y. lipolytica* also produces biosurfactants even growing in aqueous media but their detailed structure is not known [85].

Heat shock increased the levels of trehalose and changed the levels of mRNA corresponding to *YlTPS2* and *YlTPS3* but not those of *YlTPS1*. A similar lack of response of *A. nidulans tpsA* has been described [78]. The increase of mRNA corresponding to *YlTPS3* as well as the absence of trehalose in the heat shocked *Yltps3* mutant indicate an important role for the protein in the stability of the *Y. lipolytica* trehalose biosynthetic complex. While in *S. cerevisiae* the complex consists of four proteins, Tps1,Tps2, Tsl1 and Tps3, only one sequence similar to that of Tps3/Tsl1 was found in the *Y. lipolytica* database. Decrease of trehalose levels during heat shock in *S. cerevisiae* requires the disruption of both Tsl1 and Tps3 [6]. In *S. cerevisiae* different mechanisms such as transcriptional activation of some genes, stabilization of certain RNAs [86] and activation of the trehalose synthase complex [87] contribute to trehalose accumulation by heat shock. Such detailed studies are not yet available for *Y. lipolytica*. Transcriptional response to heat shock in the case of the genes of the trehalose biosynthetic pathway in *S. cerevisiae* depends on repetitions of a CCCCT stretch (STRE sequence) in their promoters [65], [87]. Function of STRE sequences in *S. cerevisiae* requires the Msn2/Msn4 proteins [64]. The corresponding gene(s) is not known in *Y. lipolytica*. Hurtado and Rachubinsky [66] observed the high sequence homology of the Zn finger domain of Mhy1 with that of Msn2/4 and showed that this protein was able to bind to STRE sequences *in vitro*. These authors reported that the levels of *MHY1* mRNA were not increased after a heat shock at 35°C. Our results show that upon a heat shock at 40°C the levels of *MHY1* mRNA increase suggesting that *MHY1* may play a role in the regulatory response to this stress. It should be noticed that the high GC content in the *Y. lipolytica* DNA [88] may cause the presence of CCCCT sequences in the promoters of several genes that have not been related with responses to stress.

*Y. lipolytica* does not grow at temperatures over 35°C. The finding that disruption of *YlTPS1* impairs growth at this limit temperature suggests that trehalose plays a protective role against the changes produced under this condition [65].

Many evidences show that in different organisms the trehalose biosynthetic pathway, in addition to its primary role, has an influence in a variety of processes that range from growth on certain substrates or temperatures, to differences in virulence in pathogens. The targets of the pathway are different depending on the organism and even closely related yeast species like *C. neoformans* and *C. gattii* show important variations in the effects caused by perturbations of that pathway [89]. The finding that in *Y. lipolytica* that separated early in evolution from other yeasts [16], the trehalose biosynthetic pathway does not regulate glycolysis suggests that this regulatory property was acquired later along yeast evolution.

## Supporting Information

Figure S1 **Growth of wild type and** ***Yltps1*** **strains.** The strains were grown as described in Materials and Methods and growth was followed measuring optical density. CJM645 (Wild type), CJM651 (*Yltps1*) and CLF279 (*Yltps1*/pCLF4). A representative curve is shown for each strain.
(TIF)

Table S1 Primers used for DNA cloning.
(DOC)

Table S2 Primers used in RT-qPCR.
(DOC)

## References

[1] Elbein AD, Pan YT, Pastuszak I, Carroll D (2003). New insights on trehalose: a multifunctional molecule.. Glycobiology.

[2] Gancedo C, Flores CL (2004). The importance of a functional trehalose biosynthetic pathway for the life of yeasts and fungi.. FEMS Yeast Res.

[3] Cabib E, Leloir LF (1958). The biosyntesis of trehalose phosphate.. J Biol Chem.

[4] Bell W, Klaassen P, Ohnacker M, Boller T, Herweijer M (1992). Characterization of the 56-kDa subunit of yeast trehalose-6-phosphate synthase and cloning of its gene reveal its identity with the product of *CIF1*, a regulator of carbon catabolite inactivation.. Eur J Biochem.

[5] De Virgilio C, Burckert N, Bell W, Jeno P, Boller T (1993). Disruption of *TPS2*, the gene encoding the 100-kDa subunit of the trehalose-6-phosphate synthase/phosphatase complex in *Saccharomyces cerevisiae*, causes accumulation of trehalose-6-phosphate and loss of trehalose-6-phosphate phosphatase activity.. Eur J Biochem.

[6] Reinders A, Burckert N, Hohmann S, Thevelein JM, Boller T (1997). Structural analysis of the subunits of the trehalose-6-phosphate synthase/phosphatase complex in *Saccharomyces cerevisiae* and their function during heat shock.. Mol Microbiol.

[7] Chen Q, Haddad GG (2004). Role of trehalose phosphate synthase and trehalose during hypoxia: from flies to mammals.. J Exp Biol.

[8] Kormish JD, McGhee JD (2005). The *C. elegans* lethal gut-obstructed *gob-1* gene is trehalose-6-phosphate phosphatase.. Dev Biol.

[9] Paul MJ, Primavesi LF, Jhurreea D, Zhang Y (2008). Trehalose metabolism and signaling.. Annu Rev Plant Biol.

[10] Gonzalez MI, Stucka R, Blazquez MA, Feldmann H, Gancedo C (1992). Molecular cloning of *CIF1*, a yeast gene necessary for growth on glucose.. Yeast.

[11] Luyten K, de Koning W, Tesseur I, Ruiz MC, Ramos J (1993). Disruption of the *Kluyveromyces lactis GGS1* gene causes inability to grow on glucose and fructose and is suppressed by mutations that reduce sugar uptake.. Eur J Biochem.

[12] Blazquez MA, Lagunas R, Gancedo C, Gancedo JM (1993). Trehalose-6-phosphate, a new regulator of yeast glycolysis that inhibits hexokinases.. FEBS Lett.

[13] Teusink B, Walsh MC, van Dam K, Westerhoff HV (1998). The danger of metabolic pathways with turbo design.. Trends Biochem Sci.

[14] Reinders A, Romano I, Wiemken A, De Virgilio C (1999). The thermophilic yeast *Hansenula polymorpha* does not require trehalose synthesis for growth at high temperatures but does for normal acquisition of thermotolerance.. J Bacteriol.

[15] Petit T, Gancedo C (1999). Molecular cloning and characterization of the gene *HXK1* encoding the hexokinase from *Yarrowia lipolytica*.. Yeast.

[16] Dujon B (2006). Yeasts illustrate the molecular mechanisms of eukaryotic genome evolution.. Trends Genet.

[17] Morin M, Monteoliva L, Insenser M, Gil C, Dominguez A (2007). Proteomic analysis reveals metabolic changes during yeast to hypha transition in *Yarrowia lipolytica*.. J Mass Spectrom.

[18] Holz M, Forster A, Mauersberger S, Barth G (2009). Aconitase overexpression changes the product ratio of citric acid production by *Yarrowia lipolytica*.. Appl Microbiol Biotechnol.

[19] Kamzolova SV, Shishkanova NV, Morgunov IG, Finogenova TV (2003). Oxygen requirements for growth and citric acid production of *Yarrowia lipolytica*.. FEMS Yeast Res.

[20] Madzak C, Gaillardin C, Beckerich JM (2004). Heterologous protein expression and secretion in the non-conventional yeast *Yarrowia lipolytica*: a review.. J Biotechnol.

[21] Beopoulos A, Cescut J, Haddouche R, Uribelarrea JL, Molina-Jouve C (2009). *Yarrowia lipolytica* as a model for bio-oil production.. Prog Lipid Res.

[22] Sakai Y, Oku M, van der Klei IJ, Kiel JA (2006). Pexophagy: autophagic degradation of peroxisomes.. Biochim Biophys Acta.

[23] Flores CL, Martinez-Costa OH, Sanchez V, Gancedo C, Aragon JJ (2005). The dimorphic yeast *Yarrowia lipolytica* possesses an atypical phosphofructokinase: characterization of the enzyme and its encoding gene.. Microbiology.

[24] Hirai M, Tanaka A, Fukui S (1975). Difference in pyruvate kinase regulation among three groups of yeasts.. Biochim Biophys Acta.

[25] Le Dall M, Nicaud J, Treton BY, Gaillardin CM (1996). The 3-phosphoglycerate kinase gene of the yeast *Yarrowia lipolytica* de-represses on gluconeogenic substrates.. Curr Genet.

[26] Flores CL, Gancedo C (2005). *Yarrowia lipolytica* mutants devoid of pyruvate carboxylase activity show an unusual growth phenotype.. Eukaryot Cell.

[27] Jardon R, Gancedo C, Flores CL (2008). The gluconeogenic enzyme fructose-1,6-bisphosphatase is dispensable for growth of the yeast *Yarrowia lipolytica* in gluconeogenic substrates.. Eukaryot Cell.

[28] Arisan-Atac I, Wolschek MF, Kubicek CP (1996). Trehalose-6-phosphate synthase A affects citrate accumulation by *Aspergillus niger* under conditions of high glycolytic flux.. FEMS Microbiol Lett.

[29] Wolschek M, Kubicek CP, Kristiansen B, Linden J, Mattey M (1998). Biochemistry of citric acid accumulation by *Aspergillus niger*.. Citric acid biotechnology.

[30] Gaillardin CM, Charoy V, Heslot H (1973). A study of copulation, sporulation and meiotic segregation in *Candida lipolytica*.. Arch Mikrobiol.

[31] Yarrow D, Kurtzman CP, Fell JW (1998). Methods for the isolation maintenance and identification of yeasts.. The yeasts: A taxonomic study.

[32] Lopez MC, Nicaud JM, Skinner HB, Vergnolle C, Kader JC (1994). A phosphatidylinositol/phosphatidylcholine transfer protein is required for differentiation of the dimorphic yeast *Yarrowia lipolytica* from the yeast to the mycelial form.. J Cell Biol.

[33] Nuttley WM, Brade AM, Eitzen GA, Glover JR, Aitchison JD (1993). Rapid identification and characterization of peroxisomal assembly mutants in *Yarrowia lipolytica*.. Yeast.

[34] Barth G, Gaillardin C, Wolf K (1996). *Yarrowia lipolytica*.. Nonconventional yeasts in biotechnology.

[35] Ito H, Fukuda Y, Murata K, Kimura A (1983). Transformation of intact yeast cells treated with alkali cations.. J Bacteriol.

[36] Navas MA, Cerdan S, Gancedo JM (1993). Futile cycles in *Saccharomyces cerevisiae* strains expressing the gluconeogenic enzymes during growth on glucose.. Proc Natl Acad Sci U S A.

[37] Sikorski RS, Hieter P (1989). A system of shuttle vectors and yeast host strains designed for efficient manipulation of DNA in *Saccharomyces cerevisiae*.. Genetics.

[38] Becker DM, Fikes JD, Guarente L (1991). A cDNA encoding a human CCAAT-binding protein cloned by functional complementation in yeast.. Proc Natl Acad Sci U S A.

[39] Blanchin-Roland S, Cordero Otero RR, Gaillardin C (1994). Two upstream activation sequences control the expression of the *XPR2* gene in the yeast *Yarrowia lipolytica*.. Mol Cell Biol.

[40] Wang H, Le Clainche A, Le Dall MT, Wache Y, Pagot Y (1998). Cloning and characterization of the peroxisomal acyl CoA oxidase *ACO3* gene from the alkane-utilizing yeast *Yarrowia lipolytica*.. Yeast.

[41] Gaillardin C, Ribet AM (1987). *LEU2* directed expression of beta-galactosidase activity and phleomycin resistance in *Yarrowia lipolytica*.. Curr Genet.

[42] Sherman DJ, Martin T, Nikolski M, Cayla C, Souciet JL (2009). Genolevures: protein families and synteny among complete hemiascomycetous yeast proteomes and genomes.. Nucleic Acids Res.

[43] Belinchon MM, Flores CL, Gancedo JM (2004). Sampling *Saccharomyces cerevisiae* cells by rapid filtration improves the yield of mRNAs.. FEMS Yeast Res.

[44] Wallenfels K, Sidney PC, Nathan OK (1962). [23] [beta]-Galactosidase (crystalline).. Methods in Enzymology.

[45] Kienle I, Burgert M, Holzer H (1993). Assay of trehalose with acid trehalase purified from *Saccharomyces cerevisiae*.. Yeast.

[46] Gamo FJ, Portillo F, Gancedo C (1993). Characterization of mutations that overcome the toxic effect of glucose on phosphoglucose isomerase less strains of *Saccharomyces cerevisiae*.. FEMS Microbiol Lett.

[47] Bergmeyer HU, Bergmeyer J, Grassi M, Bergmeyer HU (1987). Methods of enzymatic analysis..

[48] Conway EJ, Downey M (1950). An outer metabolic region of the yeast cell.. Biochem J.

[49] Thevelein JM, Hohmann S (1995). Trehalose synthase: guard to the gate of glycolysis in yeast?. Trends Biochem Sci.

[50] Kobayashi N, McEntee K (1993). Identification of cis and trans components of a novel heat shock stress regulatory pathway in *Saccharomyces cerevisiae*.. Mol Cell Biol.

[51] Bulman AL, Hubl ST, Nelson HC (2001). The DNA-binding domain of yeast heat shock transcription factor independently regulates both the N- and C-terminal activation domains.. J Biol Chem.

[52] Conlin LK, Nelson HC (2007). The natural osmolyte trehalose is a positive regulator of the heat-induced activity of yeast heat shock transcription factor.. Mol Cell Biol.

[53] Marchler G, Schuller C, Adam G, Ruis H (1993). A *Saccharomyces cerevisiae* UAS element controlled by protein kinase A activates transcription in response to a variety of stress conditions.. EMBO J.

[54] Moradas-Ferreira P, Costa V, Piper P, Mager W (1996). The molecular defences against reactive oxygen species in yeast.. Mol Microbiol.

[55] Gancedo JM, Clifton D, Fraenkel DG (1977). Yeast hexokinase mutants.. J Biol Chem.

[56] Herrero P, Galindez J, Ruiz N, Martinez-Campa C, Moreno F (1995). Transcriptional regulation of the *Saccharomyces cerevisiae HXK1*, *HXK2* and *GLK1* genes.. Yeast.

[57] Swanson RN, Conesa C, Lefebvre O, Carles C, Ruet A (1991). Isolation of TFC1, a gene encoding one of two DNA-binding subunits of yeast transcription factor tau (TFIIIC).. Proc Natl Acad Sci U S A.

[58] Huang Y, Hamada M, Maraia RJ (2000). Isolation and cloning of four subunits of a fission yeast TFIIIC complex that includes an ortholog of the human regulatory protein TFIIICbeta.. J Biol Chem.

[59] Goddijn OJ, Verwoerd TC, Voogd E, Krutwagen RW, de Graaf PT (1997). Inhibition of trehalase activity enhances trehalose accumulation in transgenic plants.. Plant Physiol.

[60] Müller J, Aeschbacher RA, Wingler A, Boller T, Wiemken A (2001). Trehalose and trehalase in *Arabidopsis*.. Plant Physiol.

[61] Müller J, Boller T, Wiemken A (1995). Effects of validamycin A, a potent trehalase inhibitor, and phytohormones on trehalose metabolism in roots and root nodules of soybean and cowpea.. Planta.

[62] Arguelles JC (1997). Thermotolerance and trehalose accumulation induced by heat shock in yeast cells of *Candida albicans*.. FEMS Microbiol Lett.

[63] Kandror O, Bretschneider N, Kreydin E, Cavalieri D, Goldberg AL (2004). Yeast adapt to near-freezing temperatures by STRE/Msn2,4-dependent induction of trehalose synthesis and certain molecular chaperones.. Mol Cell.

[64] Martinez-Pastor MT, Marchler G, Schuller C, Marchler-Bauer A, Ruis H (1996). The *Saccharomyces cerevisiae* zinc finger proteins Msn2p and Msn4p are required for transcriptional induction through the stress response element (STRE).. EMBO J.

[65] Ye Y, Zhu Y, Pan L, Li L, Wang X (2009). Gaining insight into the response logic of *Saccharomyces cerevisiae* to heat shock by combining expression profiles with metabolic pathways.. Biochem Biophys Res Commun.

[66] Hurtado CA, Rachubinski RA (1999). *MHY1* encodes a C2H2-type zinc finger protein that promotes dimorphic transition in the yeast *Yarrowia lipolytica*.. J Bacteriol.

[67] Neves MJ, Hohmann S, Bell W, Dumortier F, Luyten K (1995). Control of glucose influx into glycolysis and pleiotropic effects studied in different isogenic sets of *Saccharomyces cerevisiae* mutants in trehalose biosynthesis.. Curr Genet.

[68] De Silva-Udawatta MN, Cannon JF (2001). Roles of trehalose phosphate synthase in yeast glycogen metabolism and sporulation.. Mol Microbiol.

[69] Wolschek MF, Kubicek CP (1997). The filamentous fungus *Aspergillus niger* contains two “differentially regulated” trehalose-6-phosphate synthase-encoding genes, *tpsA* and *tpsB*.. J Biol Chem.

[70] Al-Bader N, Vanier G, Liu H, Gravelat FN, Urb M (2010). Role of trehalose biosynthesis in *Aspergillus fumigatus* development, stress response, and virulence.. Infect Immun.

[71] Adachi N, Lieber MR (2002). Bidirectional gene organization: a common architectural feature of the human genome.. Cell.

[72] Davila Lopez M, Martinez Guerra JJ, Samuelsson T (2010). Analysis of gene order conservation in eukaryotes identifies transcriptionally and functionally linked genes.. PLoS One.

[73] Tzvetkov M, Klopprogge C, Zelder O, Liebl W (2003). Genetic dissection of trehalose biosynthesis in *Corynebacterium glutamicum*: inactivation of trehalose production leads to impaired growth and an altered cell wall lipid composition.. Microbiology.

[74] Eastmond PJ, van Dijken AJ, Spielman M, Kerr A, Tissier AF (2002). Trehalose-6-phosphate synthase 1, which catalyses the first step in trehalose synthesis, is essential for *Arabidopsis* embryo maturation.. Plant J.

[75] Petit T, Blazquez MA, Gancedo C (1996). *Schizosaccharomyces pombe* possesses an unusual and a conventional hexokinase: biochemical and molecular characterization of both hexokinases.. FEBS Lett.

[76] Blazquez MA, Stucka R, Feldmann H, Gancedo C (1994). Trehalose-6-P synthase is dispensable for growth on glucose but not for spore germination in *Schizosaccharomyces pombe*.. J Bacteriol.

[77] Kramarenko T, Karp H, Jarviste A, Alamae T (2000). Sugar repression in the methylotrophic yeast *Hansenula polymorpha* studied by using hexokinase-negative, glucokinase-negative and double kinase-negative mutants.. Folia Microbiol (Praha).

[78] Fillinger S, Chaveroche MK, van Dijck P, de Vries R, Ruijter G (2001). Trehalose is required for the acquisition of tolerance to a variety of stresses in the filamentous fungus *Aspergillus nidulans*.. Microbiology.

[79] Wilson JE (2003). Isozymes of mammalian hexokinase: structure, subcellular localization and metabolic function.. J Exp Biol.

[80] Van Aelst L, Hohmann S, Bulaya B, de Koning W, Sierkstra L (1993). Molecular cloning of a gene involved in glucose sensing in the yeast *Saccharomyces cerevisiae*.. Mol Microbiol.

[81] Lin X, Heitman J (2005). Chlamydospore formation during hyphal growth in *Cryptococcus neoformans*.. Eukaryot Cell.

[82] Lowe RG, Lord M, Rybak K, Trengove RD, Oliver RP (2009). Trehalose biosynthesis is involved in sporulation of *Stagonospora nodorum*.. Fungal Genet Biol.

[83] Kretschmer A, Wagner F (1983). Characterization of biosynthetic intermediates of trehalose dicorynomycolate from *Rhodococcus erithropolis* grown on n-alcanes.. Biochim Biophys Acta.

[84] Vergne I, Daffe M (1998). Interaction of mycobacterial glycolipids with host cells.. Front Biosci.

[85] Amaral PF, Lehocky M, Barros-Timmons AM, Rocha-Leao MH, Coelho MA (2006). Cell surface characterization of *Yarrowia lipolytica* IMUFRJ 50682.. Yeast.

[86] Castells-Roca L, Garcia-Martinez J, Moreno J, Herrero E, Belli G (2011). Heat shock response in yeast involves changes in both transcription rates and mRNA stabilities.. PLoS One.

[87] Francois J, Parrou JL (2001). Reserve carbohydrates metabolism in the yeast *Saccharomyces cerevisiae*.. FEMS Microbiol Rev.

[88] Barth G, Gaillardin C (1997). Physiology and genetics of the dimorphic fungus *Yarrowia lipolytica*.. FEMS Microbiol Rev.

[89] Ngamskulrungroj P, Himmelreich U, Breger JA, Wilson C, Chayakulkeeree M (2009). The trehalose synthesis pathway is an integral part of the virulence composite for *Cryptococcus gattii*.. Infect Immun.

[90] Cherry JM, Ball C, Weng S, Juvik G, Schmidt R (1997). Genetic and physical maps of *Saccharomyces cerevisiae*.. Nature.

