# Supplementary figures and images for: Disruption of Yarrowia lipolytica TPS1 Gene Encoding Trehalose-6-P Synthase Does Not Affect Growth in Glucose but Impairs Growth at High Temperature
Source: PLoS One. 2011 Sep 12;6(9):e23695. doi: 10.1371/journal.pone.0023695 (PMC3171402; doi:10.1371/journal.pone.0023695)

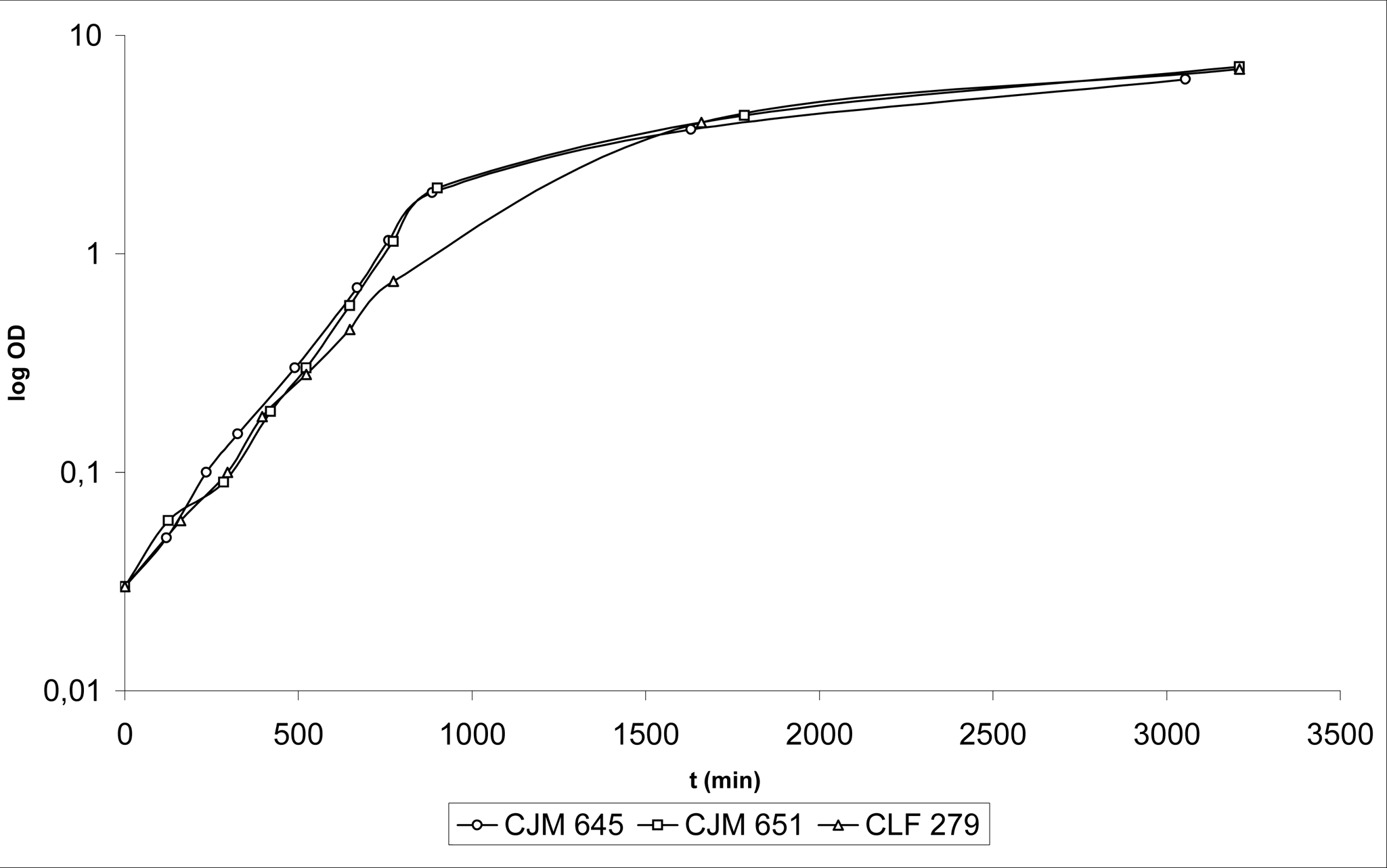

Supplement: Figure S1 — Growth of wild type and Yltps1 strains. The strains were grown as described in Materials and Methods and growth was followed measuring optical density. CJM645 (Wild type), CJM651 (Yltps1) and CLF279 (Yltps1/pCLF4). A representative curve is shown for each strain. (TIF) [file pone.0023695.s001.tif]
