# Supplementary material for: Disruption of Yarrowia lipolytica TPS1 Gene Encoding Trehalose-6-P Synthase Does Not Affect Growth in Glucose but Impairs Growth at High Temperature
Source: PLoS One. 2011 Sep 12;6(9):e23695. doi: 10.1371/journal.pone.0023695 (PMC3171402; doi:10.1371/journal.pone.0023695)
Supplement: Table S1 — Primers used for DNA cloning. (DOC) [file pone.0023695.s002.doc]

| Primer number | Target gene | Sequence |
| --- | --- | --- |
| 1000 | YALI0E14685/*YlTPS1* | GCCACATAGGGCAATGCG |
| 1001 | YALI0E14685/*YlTPS1* | CATTACCCTGTTATCCCTACCACGACATGACGCCCCTTG |
| 1002 | YALI0E14685/*YlTPS1* | CTAGGGATAACAGGGTAATGCATTGGGGCGAGGCCTTTG |
| 1003 | YALI0E14685/*YlTPS1* | GGGTCCGATGAGATCG |
| 1004 | YALI0E31086/*YlTPS3* | GCTTTAGTTGACATAGTAGC |
| 1005 | YALI0E31086/*YlTPS3* | CGAACGCGACACGGTGTTGA |
| 1006 | YALI0E31086/*YlTPS3* | CATCATGACGTTAATTGTGGTGTCATTATTCCTTCCTTACACCATC |
| 1007 | YALI0E31086/*YlTPS3* | AGAGCTGACTAAGTCTACGGCG |
| 1008 | YALI0D15598/*YlNTH1* | ccccagattgtacaaccttccc |
| 1009 | YALI0D15598/*YlNTH1* | CAACGGTACAAGTATGGACCTC |
| 1010 | *YlTPS1* Promoter | CCGCGGTCCAGATCGGAAATAGT |
| 1011 | *YlTPS1* Promoter | GGGATCCGGCAGACGGTTCGATATGAC |
| 1012 | *ScTPS1* | GCAGGCTAACAAACTAGGTACTC |
| 1013 | *ScTPS1* | GATCGTCTCATTTGCATCGGG |
