# Supplementary material for: Disruption of Yarrowia lipolytica TPS1 Gene Encoding Trehalose-6-P Synthase Does Not Affect Growth in Glucose but Impairs Growth at High Temperature
Source: PLoS One. 2011 Sep 12;6(9):e23695. doi: 10.1371/journal.pone.0023695 (PMC3171402; doi:10.1371/journal.pone.0023695)
Supplement: Table S2 — Primers used in RT-qPCR. (DOC) [file pone.0023695.s003.doc]

| Primer name | Target gene | Primer sequence |
| --- | --- | --- |
| 5’-YlTPS1 | YALI0E14685 | AGCTGGTCGGACGTATCAAC |
| 3’-YlTPS1 | YALI0E14685 | TCGCGTAGACGACACGAAAC |
| 5’-YlTPS2 | YALI0D14476 | CGCCAGTGCATCATTACACC |
| 3’-YlTPS2 | YALI0D14476 | CATGCCATCACGAACCGAAG |
| 5’-YlTPS3 | YALI0E31086 | TCTCACTCTGGCCCTTGAAC |
| 3’-YlTPS3 | YALI0E31086 | TAGGCTCGGCCTTATGGAAC |
| 5’-YlARP4 | YALI0F27533 | CGCCAATCTGCCACTGCCCA |
| 3’-YlARP4 | YALI0F27533 | CAGCACGGCTCCGTCAGCAT |
| 5’-YlGLK1 | YALI0E15488 | CACAGATCGAGACAGGAAAG |
| 3’-YlGLK1 | YALI0E15488 | CTACCAATAGCCGTAACCAG |
| 5’-YlHXK1 | YALI0B22308 | AGATCAAGCTGGGTAACATC |
| 3’-YlHXK1 | YALI0B22308 | GTCGAAGGCTCCATACTCGC |
| 5’-YlNTH1 | YALI0D15598 | CTCGAGCACGGGTCTGTGGC |
| 3’-YlNTH1 | YALI0D15598 | GTCATGTCGGGCTTGTAGGC |
| 5’-YlMCK1 | YALI0D20966 | CGGGTTCTGCTGGACAAGAG |
| 3’-YlMCK1 | YALI0D20966 | AGTGCCCGGTACACGGTTTC |
| 5’-YlMHY1 | YALI0B21582 | TCCGTTATCTCGCCATCTCC |
| 3’-YlMHY1 | YALI0B21582 | AGCTCGTCGTGTGCATACTC |
| 5’-YlHSF1 | YALI0E13948 | GTGGCAGCATGCAATCCAAC |
| 3’-YlHSF1 | YALI0E13948 | TCTGTTGCAGCTCGTCCATC |
